# Running Injury Paradigms and Their Influence on Footwear Design Features and Runner Assessment Methods: A Focused Review to Advance Evidence-Based Practice for Running Medicine Clinicians

**DOI:** 10.3389/fspor.2022.815675

**Published:** 2022-03-09

**Authors:** Cristine Agresta, Christina Giacomazzi, Mark Harrast, Jessica Zendler

**Affiliations:** ^1^Department of Rehabilitation Medicine, University of Washington, Seattle, WA, United States; ^2^Zendler Scientific, Tacoma, WA, United States

**Keywords:** running, shoes, biomechanics, injury, sports medicine, footwear

## Abstract

Many runners seek health professional advice regarding footwear recommendations to reduce injury risk. Unfortunately, many clinicians, as well as runners, have ideas about how to select running footwear that are not scientifically supported. This is likely because much of the research on running footwear has not been highly accessible outside of the technical footwear research circle. Therefore, the purpose of this narrative review is to update clinical readers on the state of the science for assessing runners and recommending running footwear that facilitate the goals of the runner. We begin with a review of basic footwear construction and the features thought to influence biomechanics relevant to the running medicine practitioner. Subsequently, we review the four main paradigms that have driven footwear design and recommendation with respect to injury risk reduction: Pronation Control, Impact Force Modification, Habitual Joint (Motion) Path, and Comfort Filter. We find that evidence in support of any paradigm is generally limited. In the absence of a clearly supported paradigm, we propose that in general clinicians should recommend footwear that is lightweight, comfortable, and has minimal pronation control technology. We further encourage clinicians to arm themselves with the basic understanding of the known effects of specific footwear features on biomechanics in order to better recommend footwear on a patient-by-patient basis.

## Introduction

Many runners seek health professional advice regarding footwear recommendations to reduce injury risk. Footwear design and its ability to positively influence running biomechanics and injury risk remains under debate. Nonetheless, health professionals continue to make recommendations to patients about which running footwear to select to reduce injury risk. Moreover, recent studies have found that clinicians—as well as runners—have ideas about risk factors, including footwear, that are not scientifically supported (Rothschild, 2012; Saragiotto et al., 2014b; Dhillon et al., 2020; Wolthon et al., 2020). We expect that several factors account for the lack of application of current evidence when recommending footwear to patients. Firstly, clinicians may be unaware of the complex construction of running footwear, specifically the subcomponents of midsole construction. Secondly, high-quality footwear research is often published in niche peer-reviewed journals (e.g., Footwear Science) that do not commonly reach clinical readers or in journals that sit behind a paywall and cannot be readily accessed by clinicians practicing outside academic institutions. Finally, there is a lack of knowledge regarding the conceptual paradigms that underpin current and past footwear recommendations, the research evidence (or lack thereof) in support of them, and how they manifest in runner assessment methods and key footwear design features. We contend that clinicians tasked with matching footwear to a specific running would benefit from an understanding of how running shoe design is tightly connected to theoretical paradigms of running injury etiology and how these factors together (paradigm and design) directly influence runner assessment and footwear recommendation methodologies.

The purpose of this review is to update clinical readers on the state-of-the science for assessing runners and recommending footwear. Our goal is to advance evidence-based practices of running medicine clinicians related to footwear assessment and recommendations. To do this, we provide a basic overview of running footwear construction, highlighting the key features thought to influence running biomechanics. Subsequently, we review the main paradigms that have dictated footwear construction, and in turn, footwear recommendations over the last half-century to illustrate how concepts about risk factors for running-related injuries influence footwear design and clinical recommendations. Historically, these paradigms have focused on using proper footwear-runner matching to reduce the incidence of running-related injuries or biomechanics linked to injury risk. We briefly discuss the performance-driven features of running footwear and conclude the review with a discussion of practical implications for applying these paradigms to footwear recommendation, considerations for incorporating performance goals into footwear prescription, and areas for future research.

## Footwear Construction

Shoe technology is intended to positively influence performance, feel, or injury risk. Understanding footwear construction and its implications on running biomechanics can improve clinical reasoning and patient/athlete communication about appropriate running footwear selection. Running footwear construction has gone through a large overhaul in the past 55 years, with a particularly rapid evolution in the last decade (Davis, 2014). Running shoes have cycled from the low structure and low cushioning of the Onitsuka Tiger shoes that emerged in 1964 as the first cushioned running shoes, to the highly structured and cushioned shoes that dominated much of the 80's−2000's, and now have swung back toward less structure coupled with a wide range of (from no to significant) cushioning. These large swings in shoe construction have paralleled changes in paradigms around the relation between running biomechanics and injury or performance, which we will discuss in subsequent sections.

**Figure 1:**
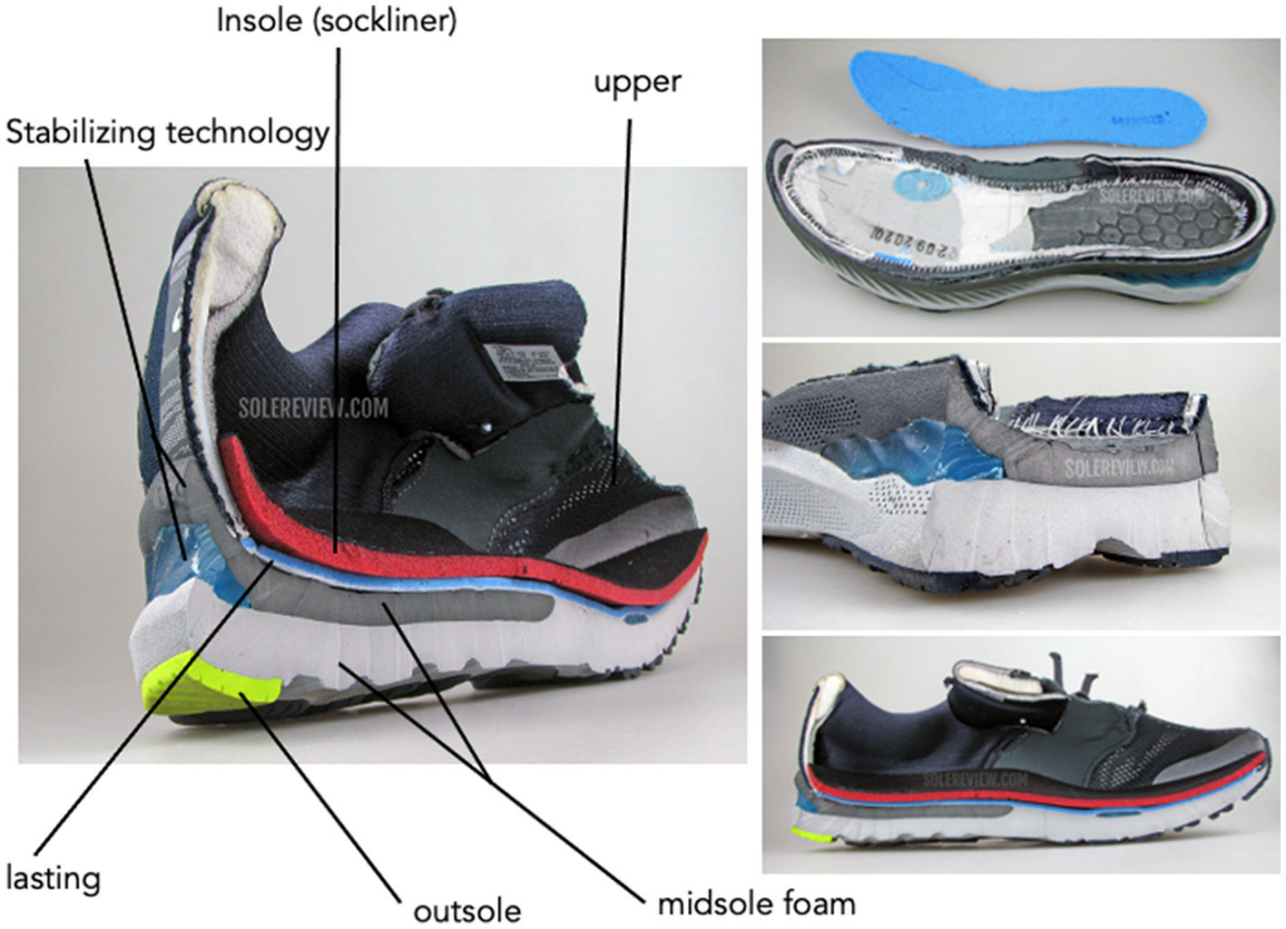
Dissections of Asics Gel Nimbus 23. Images obtained with written permission from solereview.com.

Regardless of the paradigm driving footwear construction, all running shoes have two major components: the upper and the sole. The upper is the component of the shoe that covers the dorsum and heel of the foot. Upper construction includes the lacing system and heel counter. The upper construction influences breathability and fit, partially through the lacing system. To date, there is very little research about how the components of the upper influences running injury or performance (Hoitz et al., 2020; Sun et al., 2020). The sole is the component of the shoe that covers the plantar surface of the foot and interacts with the ground. It is typically subdivided into three parts: (1) insole, (2) midsole, and (3) outsole (Figure 1). Since much of the technology differences and changes among running shoes occur within the midsole, the influence of midsole construction on running biomechanics, injury risk, and/or performance has received the most attention (Hoitz et al., 2020). Comparatively, little is known about how insole or outsole properties influence injury- or performance-related variables. Consequently, most of this article will focus on midsole construction and its influence on running biomechanics, acknowledging that more research is needed into upper, insole, and outsole effects on running biomechanics, injury, and performance.

**Figure 2:**
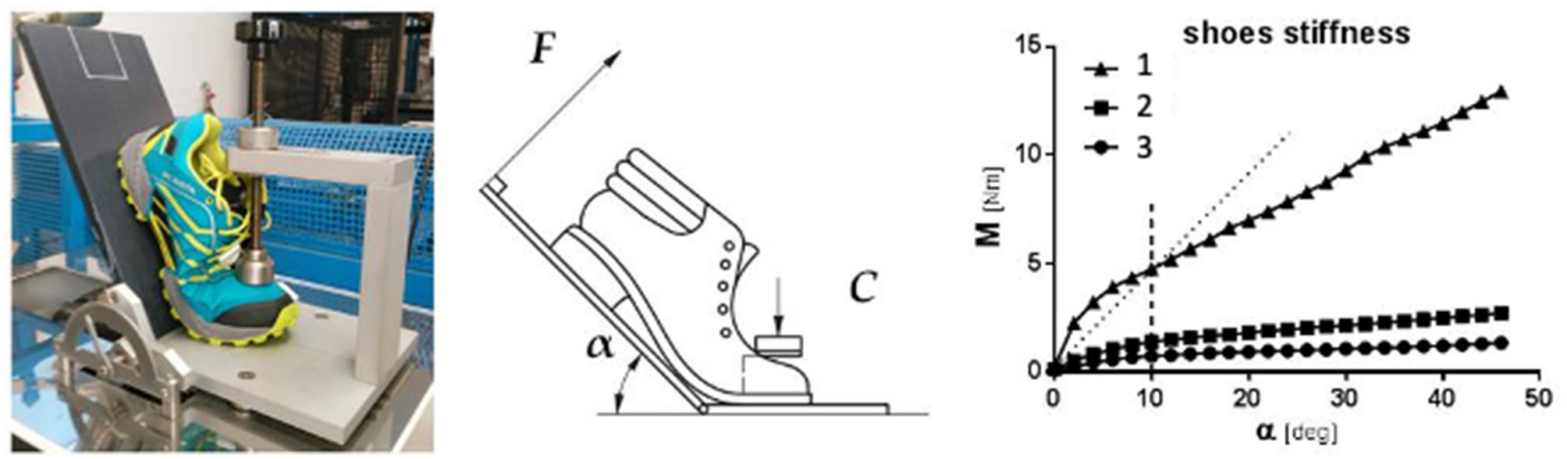
Illustration of longitudinal bending stiffness): (left) stiffness bench test and set-up (EN ISO 20344:2011; (middle) schematic representation of the stiffness test. α: bending angle; F: bending force; C: clamping force; (right) the bending angle α is reported on the x-axis, whereas the corresponding bending moment M, coming from the bending force F, is shown in the y-axis. Taken from Mistretta et al. (2018).

The midsole can be described by several major characteristics including thickness, heel-to-toe drop, motion control and stability technologies, flexibility (torsional and longitudinal bending stiffness), and material composition [e.g., type(s) and distribution of foam used]. These characteristics give rise to the thickness, bending stiffness, resiliency—sometimes referred to as the cushioning system, and overall mass of the shoe. Midsole thickness, sometimes referred to as stack height, is simply defined as the vertical height of the midsole of the shoe (Hoitz et al., 2020). The term heel-to-toe-drop typically describes the difference between the heel height and the forefoot height. Midsole longitudinal bending stiffness is the resistance to bending around a medio-lateral axis of the shoe (Figure 2). It is currently believed that bending stiffness is most heavily influenced by additives to the midsole, specifically the presence of a carbon fiber plate. However, other midsole modifications like increased thickness, midsole material selection, and the addition of flex grooves or thermoplastic polyurethane inserts are influential. Increasing midsole bending stiffness is typically associated with performance improvement (e.g., faster running speed) (Nigg et al., 2020). However, the benefit of adding a carbon fiber plate to increase midsole bending stiffness and the important characteristics of the plate, including its shape, remain under debate (Beck et al., 2020; Healey and Hoogkamer, 2020).

Midsole resiliency refers to how much energy will be returned by the midsole with each step. The type and amount of midsole materials used can alter the cushioning features of the shoe, the feeling of hardness/softness, and its energy return. Traditionally, the midsole is constructed from either ethylene vinyl acetate (EVA) or thermoplastic polyurethane (TPU). More recent running footwear (e.g., Nike Vaporfly 4%) use new technology to produce lighter, less dense, and more resilient material (Burns and Tam, 2020). These materials are suggested to maintain high cushioning and a feeling of softness while improving energy return (less energy lost with each step) and reducing shoe mass.

## Running-Related Injury Paradigms for Footwear Construction and Recommendation

A fundamental assumption in running footwear research is that shoes influence the running experience that is manifested, in part, in the running biomechanics. This is supported by studies that demonstrate immediate kinematic and kinetic differences for participants running in different types of running shoes (Tung et al., 2014; Sinclair et al., 2016a; Agresta et al., 2018; Flores et al., 2019). Conceptual paradigms for footwear recommendations expand on this assumption by creating theoretical frameworks for biomechanical risk factors for running injuries and, in turn, how specific footwear features can counteract these risk factors. To date, there are four major conceptual paradigms for running-related injury that have driven footwear design, methods of runner assessment, and footwear recommendations They represent differing and evolving schools of thought regarding injury development and will be referred herein as: (1) Pronation Control, (2) Impact Force Modification, (3) Habitual Joint (Motion) Path, and (4) Comfort Filter. The Pronation Control and Impact Force Modification paradigms share similar origins (Hintermann and Nigg, 1998) but have diverged in rationale and recommendation with time. Likewise, the newer paradigms of Habitual Joint Path and Comfort Filter emerged from the older ones and, thus, share some conceptual foundations (Nigg, 2001; Nigg et al., 2015).

The subsequent four sections will address each paradigm in (roughly) chronological order of its emergence. Each section is divided into four parts: the biomechanical rationale underlying the paradigm, the key features manipulated under this rationale, the philosophy for matching runner to shoe, and a brief summary of the research evidence related to the efficacy of footwear recommendation based on the paradigm. We caution the reader to remember that our intention is to be descriptive rather than prescriptive in order to establish a useful conceptual framework for considering specific running footwear for specific runner needs. We aim to highlight that historically the development of footwear features has evolved from conceptualization of potential risk factors for running injury and these concepts must be considered when matching footwear to runner. Our review is retrospective and thus some of the rationale and assessment methods have not been supported in subsequent research.

### Pronation Control

#### Rationale

The Pronation Control paradigm is one of the earliest and most well-known of all the footwear paradigms. Emerging in the late 1970s, footwear was seen as a possible avenue to respond to the increase in running-related injuries paralleling the rise in popularity of recreational running (Jacobs and Berson, 1986). The main premise of the Pronation Control paradigm was that excessive pronation at the subtalar joint during the stance phase of running increases stress on the anterior medial region of the knee, leading to pain and/or injury. Specifically, subtalar pronation promotes internal rotation of the tibia through joint coupling with the foot, which in turn was thought to place greater stress at the tibiofemoral and/or patellofemoral joints (Hintermann and Nigg, 1998). Since most of the injuries in recreational runners were—and still are—at the knee (van Gent et al., 2007; Tonoli et al., 2010), using footwear to prevent increased knee joint stress was a logical method to reduce knee pain and injury (Stefanyshyn et al., 2006; Liao et al., 2018).

#### Key Footwear Feature

Shoes aligning with this paradigm are constructed with “motion control” technology. Typical motion control technology involves a region of dense (harder) midsole material along the longitudinal mediolateral arch to reduce midfoot pronation during stance. Alternatively, or additionally, posting, or wedging is used at the rear portion of the shoe to reduce rearfoot motion and provide increased stability to the heel. Since pronation is a tri-planar motion of the foot and ankle complex (calcaneal/rearfoot eversion, forefoot abduction, and ankle dorsiflexion) and involves all three regions of the foot (fore-, mid-, and rear-foot), minimizing rearfoot eversion can influence pronation.

#### Runner Assessment Approach and Recommendation

Runner assessment for recommending footwear in line with the Pronation Control paradigm typically begins with evaluating static foot posture but also may include assessing dynamic foot movement during activity. Static foot posture is typically evaluated in double-leg standing and includes medial longitudinal arch height, navicular height, and amount of rearfoot eversion from subtalar neutral. Additionally, the Foot Posture Index (FPI-6) can be used clinically to manually quantify or classify foot posture (Redmond et al., 2006). The assessor may ask the runner to perform a series of single leg squats to observe the amount of change in arch/navicular height in combination with knee frontal plane position (Magrum and Wilder, 2010). If room or equipment is available, runners may be asked to run while the practitioner examines rearfoot eversion and pronation position at midstance. Typically, health care professionals use qualitative analysis (Nicola and Jewison, 2012), but recent advancements and access to video annotation software allow for quantification of rearfoot eversion angle using 2D video (Souza, 2016).

From this assessment, a health care professional would determine whether foot posture and/or motion during running was considered a risk factor for injury and needed to be controlled, in part, using shoe technology. If so, a stability or motion control shoe would be recommended based on the severity of foot posture/motion. Specifically, “low” arch height and/or “very excessive” pronation/rearfoot motion would be matched to a motion control shoe, which has significant motion control technology. A “medium” arch height and/or “excessive” pronation/rearfoot motion would be matched to a stability shoe, which has some motion control technology but less than a motion control shoe. Importantly, there is no standard accepted threshold for *excessive* pronation or medial (frontal plane) knee motion, although normative running biomechanics suggest that the rearfoot is typically in 6°-8° of calcaneal inversion at initial contact and shifts to ~6°-8° of eversion by midstance (Dicharry, 2010).

It is important to highlight that while we have laid out the original paradigm rationale and accompanying approach and recommendation, most of the current evidence does not support the underlying rationale of injury paradigm or that the key footwear feature reduces risk. Specifically, excessive pronation was not found to be a risk factor for non-specific injuries in a large prospective study of novice runners (Nielsen et al., 2014) and was not associated with specific types of running-related injuries (Chuter and Janse de Jonge, 2012). Moreover, limited evidence exists to indicate that structural alignment is a primary risk factor for injury (Wen et al., 1997, 1998; Saragiotto et al., 2014a) or that static foot posture accurately reflects dynamic foot motion during running (Behling and Nigg, 2020).

#### Current Evidence

Studies support that midsole technology can effectively alter rearfoot motion (Cheung et al., 2011). VanWoensel and Cavanagh (van Woensel and Cavanagh, 1992) tested customized shoes with the same midsole hardness and varied the degrees of rearfoot posting (10 degrees of valgus, 10 degrees of varus, and neutral shoe). Shoes with varus or valgus posting produced forced supination or pronation, respectively, when compared to running in a neutral midsole. Lilley and Dixon (2013) examined foot kinematics and knee forces in 30 females with a minimum of 3 years of running experience while running in a motion control (Adidas Supernova Sequence) and a neutral shoe (Adidas Supernova Glide). Peak rearfoot eversion and knee internal rotation moment were reduced in the motion control shoe compared to the neutral shoe. Butler et al. (2006) studied several measures of rearfoot motion, tibial motion, and loading in 20 low-arched and 20 high-arched recreational runners. Arch height was classified with the Arch Height Index Measurement System. All participants ran in a motion control shoe (New Balance 1122) and a neutral shoe (New Balance 1022). While the motion control and neutral shoes elicited significant differences in most of the biomechanical parameters, only instantaneous vertical loading rate was significantly different between low- and high-arch runners. Thus, study authors questioned the validity of matching an arch type to a shoe type to reduce injury risk.

Several studies have examined the incidence of injury following prescription of running shoes based on foot posture assessment. Two large prospective studies (Knapik et al., 2010, 2014) were performed on military cadets who were assigned one of three running shoes (motion control, stability, neutral) based on their arch height (low, medium, high). Arch height was measured by two independent classifiers who evaluated an image created from standing on a reflective mirror and were classified into three categories (high, medium, or low arches). Assigning shoes based on arch type did not significantly reduce the rate of injuries during 12 weeks of basic training for male or female military recruits (Knapik et al., 2010, 2014). Ryan et al. (2011) found that female recreational runners who were randomly assigned motion control shoes incurred more missed days (79 days) due to pain during a 13-week half marathon training program than those assigned a neutral (64 days) or stability (51 days) shoe. Likewise, Nielsen et al. (2014) did not find significant differences in injury risk among 927 novice runners with varying arch types who trained in neutral shoes. Malisoux et al. (2016) found contrasting results when randomly assigning either a neutral or motion control shoe to a group of 372 recreational runners. During 6-months of observation, sixty (of 185) runners in the neutral shoe group reported an injury compared to 33 (of 187) in the motion control group. However, further research is needed to substantiate these findings as twice as many runners in the motion control group were censored in analysis with both groups having relatively equal numbers of injury free runners (91 in the neutral shoe group compared to 96 in the motion control shoe group). Together these findings suggest that recommending footwear from static foot posture assessment or degree of rearfoot eversion during stance phase of running is not currently supported in most cases and that reducing foot motion through motion control shoes may even be injurious.

### Impact Force Modification

#### Rationale

**Figure 3:**
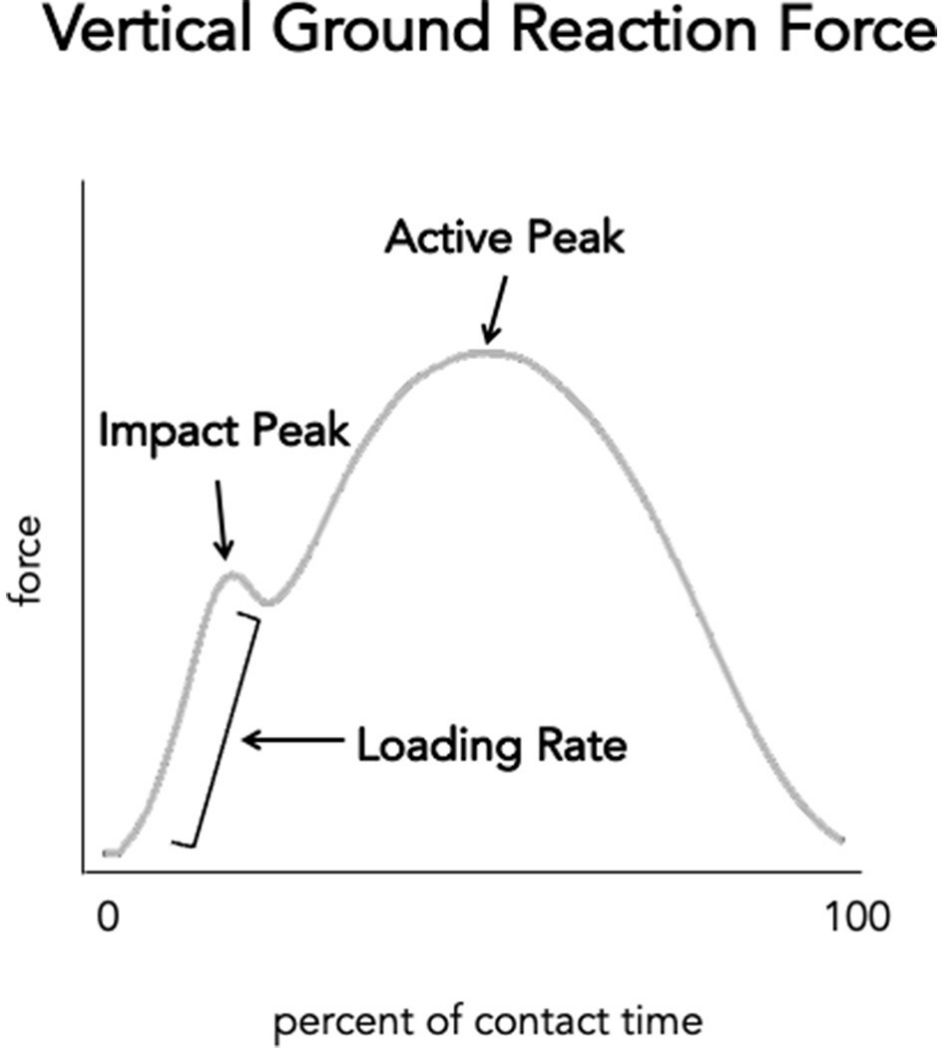
Schematic of vertical ground reaction force curve representing the stance (contact) phase of running. The y-axis represents the magnitude of vertical ground reaction force typically measured in Newtons or in Bodyweights. The x-axis represents percent of stance (or contact time). Please note that there is some variation in methods to identify initial contact and toe-off—which define stance phase—the vertical impact peak and active peak as well as the calculation for vertical loading rate.

The Impact Force Modification Paradigm theorizes that excessive forces imparted on the body during the impact phase of running (i.e., landing and loading response phases) contribute to injury. Primarily two biomechanical features, the impact peak and the loading rate (slope of the rising portion) of the vertical ground reaction force (Figure 3), have been targeted under this paradigm. These features were selected because they occur during the loading phase of stance and were thought to correlate with larger (injurious) body loads. While the impact peak itself has not been well-correlated to injury, a larger (steeper) vertical ground reaction force loading rate has been suggested as a risk factor for injury in distance runners (Hreljac, 2004; Zadpoor and Nikooyan, 2011; van der Worp et al., 2016). However, recent prospective studies call into question the evidence supporting this association, particularly as a global risk factor for running-related injuries (Ceyssens et al., 2019). The presence of an impact peak also can provide information about the foot strike pattern. A visible impact peak is typically associated with rearfoot striking (Lieberman et al., 2010; Daoud et al., 2012), whereas impact peak is typically less pronounced or absent in non-rearfoot striking. This phenomenon becomes important to understanding the development of minimalist footwear, which aims to reduce impact by promoting a non-rearfoot strike pattern, and maximalist footwear that aims to reduce impact forces with increased midsole thickness.

#### Key Footwear Feature

Historically, midsole cushioning has been the primary footwear feature targeted by this paradigm. Initial studies found that vertical ground reaction force loading rates were less steep when runners wore a more cushioned shoe (Clarke et al., 1983). Thus, since the majority of recreational runners are rearfoot strikers (de Almeida et al., 2015), meaning they contact the ground first with their heel, a cushioned heel became the target feature to mitigate injury (Hintermann and Nigg, 1998). Conventional running shoes typically did not vary in the amount of thickness until mid 2000s. Prior to this time most changes in midsole cushioning were driven by selecting materials that absorbed more energy at impact. Beginning in the mid 2000s alterations to the midsole thickness also have been employed to alter cushioning properties. Increased thickness also allows for variations in the ratio of rearfoot to forefoot thickness (heel-to-toe drop) and the ability to add technology to the midsole (e.g., carbon fiber plates). These allowances have been targeted to alter foot strike pattern and promote a shift of the ground reaction force anteriorly during stance (Nigg et al., 2021), respectively.

#### Runner Assessment and Recommendation

The most common assessment method to estimate injury risk is still observation of running form. Traditionally, measuring ground reaction forces to calculate loading rates and estimate joint resultant forces and moments required expensive, laboratory-based force-, or pressure-sensing devices like platforms and instrumented treadmills. More recently, wearable accelerometers have been used to measure peak positive tibial acceleration as a surrogate for loading rate (Henning and LaFortune, 1991; Van den Berghe et al., 2019) while force- or pressure-sensing insoles have been used to directly measure vertical ground reaction force during running. However, these wearable devices do not provide sufficient data to calculate joint kinetics. Foot strike pattern, foot inclination angle (the angle between the foot and the horizontal in the sagittal plane), step rate, and stride length are considered kinematic correlates to ground reaction or joint forces (Heiderscheit et al., 2011; Lenhart et al., 2014a,b; Wille et al., 2014; Souza, 2016). Runners who rearfoot strike tend to have low step rates, use longer steps, and run with higher magnitudes of peak vertical ground reaction force, loading rates, peak braking forces, and potentially larger lower extremity joint forces than non-rearfoot strikers. Following the paradigm, clinicians would recommended a more cushioned running shoe to these runners to mitigate the higher repetitive forces. Importantly, there are no quantitative cut-points or thresholds used to determine a “risky” kinematic measurement. Moreover, as stated above, strong evidence to support the underlying kinetic measure to which these kinematic features are associated is lacking (Ceyssens et al., 2019).

#### Current Evidence

Currently, there is not sufficient evidence to support that increasing cushioning via changes in midsole material reduces external vertical ground reaction forces (Baltich et al., 2013, 2015; Addison and Lieberman, 2015; Malisoux et al., 2021), which is the rationale behind the paradigm. It is important to note that the validity of using the impact peak of the vertical ground reaction force to evaluate the effect of shoe cushioning has been questioned Shorten and Mienjtjes (Shorten and Mientjes, 2011). Shorten and Mienjtjes demonstrated that the effect of cushioning on reducing impact forces generated under the heel was pronounced when specific analyses (frequency-domain rather than traditional time-domain) were applied. However, studies using in-sole pressure sensors have found differences in impact across cushioning levels (Kersting and Bruggemann, 2006; Dixon, 2008; Shorten and Mientjes, 2011). With respect to injury, more research is needed to determine the influence of cushioning on injury risk as only two studies have examined this relationship and results are mixed (Theisen et al., 2014; Malisoux et al., 2020), with Malisoux et al. (2020) finding that only lighter runners experience the protective effect of cushioning.

The minimalist shoe movement emerged as a variation of the impact modification paradigm, beginning in the mid 2000s. It was a response to the insufficiency of cushioned running shoes to reduce impact forces or injuries. This movement was fuelled by the rapidly rising interest in barefoot running around the same time (Rothschild, 2012; Davis, 2014). Minimalist shoes are designed to be thin, have a flexible midsole, and offer minimal to no arch stabilizing technology. Minimalist shoes are intended to permit free motion of the foot to achieve as close to a *barefoot* style of running as possible, while still providing protection from glass, debris, and rough road surfaces. The rationale for using minimalist shoes for impact force modification is that a barefoot running style is associated with a forefoot strike pattern, which eliminates the appearance of an impact peak on the vertical ground reaction force curve (Lieberman et al., 2010). A forefoot strike pattern has also been associated with lower magnitude and loading rate of impact forces in forefoot compared to rearfoot strikers (Almeida et al., 2015; Xu et al., 2021). The magnitude of midsole thickness is the main feature distinguishing minimalist shoes from each other and traditional footwear. Esculier et al. (2015) developed a standardized method for classifying minimalist shoes called the Minimalist Index Rating Scale. It is important to note that minimalist shoes do not always lead to a significant shift in footstrike pattern. Not all rearfoot strikers transition to a forefoot strike pattern when footwear is reduced (Willson et al., 2014; Tam et al., 2016). Moreover, runners who rearfoot strike in minimalist shoes tend to demonstrate higher peak ground reaction forces and vertical loading rates compared to conventional shoes (Paquette et al., 2013; Willson et al., 2014; Willy and Davis, 2014; Rice et al., 2016). Thus, debate continues about whether foot strike pattern (Lieberman et al., 2010; Lieberman, 2012; Shih et al., 2013), type of running shoe (Giandolini et al., 2013; Rice et al., 2016), or a combination of the two is best to reduce impact forces.

However, studies examining the influence of minimalist shoes on injury or running biomechanics related to injury do not support initial claims. Specifically, higher vertical ground reaction force loading rates and impact peaks were found when running in minimalist compared to conventional shoes (Sinclair et al., 2016a; Agresta et al., 2018; Jandová et al., 2019). Tibial acceleration, which has been suggested as an in-field measure for vertical loading rate (Van den Berghe et al., 2019), also was higher when running in minimalist compared to conventional shoes (TenBroek et al., 2014; Agresta et al., 2018). Moreover, there is evidence to suggest that switching from traditional to minimalist shoes may contribute to short-term (up to 12 weeks) foot and lower leg pain or injury (Ryan et al., 2014; Agresta et al., 2018) but may result in plantar-flexor strength gains in the longer-term (20 weeks) (Fuller et al., 2019). Body mass also appears to modulate the relation between cushioning and injury risk as heavier runners seems to be at greater risk of injury in minimalist shoes (Fuller et al., 2017). Fuller et al. (2017) found an influence of weekly training distance (>35 km/week) on injury risk in minimal shoes. However, this finding may relate more to a global running consideration rather than a runner-shoe interaction. Previous research (Rasmussen et al., 2013) has found injury risk increases at a similar weekly volume (30 km/week) when footwear wasn't controlled, and material testing has demonstrated deterioration of the functional properties of shoes after only 1,000 km of use (Hennig, 2011).

Maximalist shoes are another recent evolution of the Impact Force Modification Paradigm and a counterpart to minimalist shoes. Like minimalist shoes, midsole thickness is the primary distinguishing feature from traditional shoes. However, unlike minimalist shoes, no standardization currently exists for classifying maximalist shoes. Generally, shoes having more than 20 mm midsole thickness and minimal support technology are considered maximalist shoes. Less research exists on maximalist shoes compared to minimalist shoes. Maximalist shoes were first adopted by those who felt the extra cushioning protected against joint stress but otherwise wanted minimal support structure to promote a “natural” gait pattern. While the increase in midsole thickness of a maximalist shoe does not seem to reduce vertical ground reaction force (Agresta et al., 2018; Kulmala et al., 2018) or joint resultant forces (Sinclair et al., 2016b; Chan et al., 2018), it does appear to be less likely to cause running-related pain or time-loss from running compared to minimalist shoes (Agresta et al., 2018), suggesting there may be some protective value in shoes having large midsole thickness.

### Habitual Joint (Motion) Path

#### Rationale

The main premise of the Habitual Joint (Motion) Path Paradigm is that each runner has a unique trajectory for the motion of their joints (Nigg et al., 2015). The magnitude of the joint motion may fluctuate, but the trajectory, or path, is stable. Alterations in footwear that resist or do not allow for skeletal movement along this path may increase tissue stress—either from a deviation from their pathway or from increased muscle activity to keep their individual habitual joint path—which in turn increases the risk of injury (Nigg, 2001; Enders et al., 2013; Willwacher et al., 2020). Originally called the Preferred Movement Path, this paradigm has since been updated and renamed the “habitual joint (motion) path,” adding that joint motion takes the path of least resistance due to an individual's anatomy and passive tissue properties (Trudeau et al., 2019).

Highly controlled studies examining segment-to-shoe motion support the concept that runners have an individual joint path of motion. Nigg (2010) used bone pins in the femur, tibia, and calcaneus to measure skeletal motion during running and found minimal variation in skeletal movement despite changes in footwear. Additional studies using skin or shoe mounted markers to measure limb motion while manipulating shoe inserts have illustrated similar results (Eng and Pierrynowski, 1994; Nawoczenski et al., 1995). Importantly, both the Pronation Control and Impact Force Modification Paradigms focus on a specific biomechanical parameter with generalized norms for how footwear should influence it. The Habitual Joint (Motion) Path Paradigm moves away from generalizations and toward a more runner-specific recommendation method.

#### Key Footwear Feature

Since, by definition, habitual joint motion patterns are subject-specific, there is no specific key feature associated with this paradigm. However, an example may be minimal arch support that allows for the preferred movement of the foot into pronation rather than a medial post that may promote a more supinated position of the foot (opposite of the preferred path).

#### Runner Assessment Approach and Recommendation

The Habitual Joint (Motion) Path Paradigm attempts to match runners to footwear that minimizes variability, or deviation, away from their habitual joint motion path. Currently, there are no standardized or common clinical tests to assess variability of or deviation from the habitual path. Trudeau et al. (2019) developed a field method to assess deviation in order to recommend footwear. The field method evaluates a runner's baseline habitual motion path by measuring lower-limb kinematics during double-legged half squats. The half squat position was chosen because it induces lower-limb flexion/extension movement similar to running but with less than half the force on the limb. Subsequently, participants completed a short run in a sock shoe, which has no footwear technology but a minimum level of cushioning to promote a “normal” gait pattern. Knee and ankle kinematics were compared between the squat and the run to determine whether the runner is a “high deviator” or “low deviator.” High deviators displayed large differences between the half-squat and run, and vice versa for the low deviators. Finally, they were tested with selected running shoes. Running shoes were deemed as *appropriate* if they decreased the difference between running and half-squat kinematics and *inappropriate* if they increased this deviation.

#### Current Evidence

A few studies (Fuller et al., 2016; Schrödter et al., 2016; Weir et al., 2020a) have investigated the Habitual Joint (Motion) Path paradigm by examining the influence of running shoes on different measures of movement variability. In contrast to Pronation Control and Impact Force Modification, which relied on joint motions or forces measured at specific time points (e.g., initial contact and midstance) to test the effect of footwear, the Habitual Joint (Motion) Path requires a more wholistic calculation of how either whole body or select body segments are adapting across the entire gait cycle or multiple gait cycles. Testing the consistency of movement patterns—or its opposite, variability—is one such wholistic approach to studying movement.

Schrödter et al. (2016) defined “footwear-related variability” as the influence of shoes to force an individual outside their habitual motion path. They calculated the similarity of a runner's kinematical and kinetical patterns across a variety of footwear conditions. Runners with high footwear-related variability had patterns that changed more across footwear conditions. Based on their findings, the authors considered these runners to be more sensitive to running footwear, less able to maintain their habitual motion path, and at higher risk of injury compared to runners with low footwear-related variability. Runners having higher footwear-related variability also tended, albeit with weak association, to have lower peak knee adduction angle and moment, initial contact and peak hip external rotation angle, and peak ankle external rotation moment, suggesting a difference in running style between those runners who are more and less sensitive to footwear. Additionally, footwear-related variability was significantly correlated with gender, weight, and some measures of sagittal plane strength, which the authors posited could suggest female, lighter, and weaker runners are less able to maintain their habitual motion path.

Coordination variability is another biomechanical measure that has been used to explore this paradigm. In this case, coordination refers to the segment and/or joint pattern that is used to accomplish a given movement task, while variability refers to breadth of coordination patterns used by the individual to achieve the task. Coordination variability is considered indicative of the amount of flexibility of the motor control system and may have an optimal range, above or below which could lead to injury (Hamill et al., 2012). Weir et al. (2020b) examined coordination variability of the lower limb during a prolonged fatiguing running in a group of male recreational runners. Runners exhibited higher coordination variability for three of the seven measures in neutral shoes compared to stability shoes. However, it is not clear whether increased coordination variability in the neutral shoe represents a negative deviation from the habitual motion path in response to fatigue or a positive adaptation to fatigue that is not necessary or not possible in the stability shoe.

Fuller et al. (2016) examined the similarity of stride time from one stride to the next in traditional and minimalist footwear and found that there were generally no differences between shoes. The only exception was a subset of runners who spontaneously switched from their normal rearfoot pattern to a midfoot pattern when introduced to the minimalist shoe. This group reduced consistency, as measured by the long-range correlation of stride time, at the fastest speed tested. However, difference in variability between footfall groups did not reach significance. Long-range correlations indicate how similar a pattern is over time, with an alpha of 0.50 indicating that strides are uncorrelated. Debate exists as to whether a reduction in long-range correlations indicates a positive or negative response (Dingwell and Cusumano, 2010; Van Orden et al., 2011; Cusumano and Dingwell, 2013). In this study, the decrease in alpha combined the increase in coefficient of variation, as was the case with the runners who switched to a midfoot strike, suggest that these runners needed to make more corrections in stride and, thus, are potentially less able to adapt their gait pattern to novel running footwear and potentially more susceptible to injury.

To our knowledge, no studies have directly tested the effect of matching footwear to minimize biomechanical variability and/or deviation from a specific motion path on running related injury. However, one study (Willwacher et al., 2020) suggested that increased time outside one's habitual motion path was associated with tissue-related changes in the knee joint. In this study of 12 healthy recreational runners, medial femur, medial tibia, and patella cartilage volume reductions were larger after 75 min of running in a shoe that increased a runner's deviation from their habitual joint path compared to one that reduced the deviation.

### Comfort Filter

#### Rationale

The Comfort Filter Paradigm, proposed by Nigg et al. (2015), posits that a runner intuitively selects a shoe that is biomechanically optimal based on comfort. This emerging viewpoint suggests that subjective comfort is the most important factor for selecting running footwear to reduce injury risk (Nigg et al., 2015). One hypothesis about why comfortable shoes are less injurious is that increased impact forces, which are expected to increase injury risk, lead to more soft tissue vibrations. These vibrations must be counteracted by muscle activation, also referred to as muscle tuning, which feels uncomfortable and requires higher energy expenditure (Nigg, 2001). The hypothesis that comfort is linked to reduced injury risk was partially developed from research on the effect of insoles on movement patterns and injury in soldiers. Mundermann et al. (2001) provided six different insoles to 206 military training personnel without lower extremity injuries and asked them to assess comfort of the insoles. The test group received the insole that they rated most comfortable and used it for the next 4 months. They were compared with a control group of soldiers with no insoles who were exposed to the same military training. The test group had 53% fewer lower-extremity injuries than the control group. Interestingly, five out of six insoles were selected as most comfortable at a similar rate, which suggests that comfort was linked to individual-specific rather than insole-specific factors.

#### Key Footwear Feature

Since this paradigm is runner-specific and relies on subjective perception, there is not a key feature of shoe design associated with it. However, one could argue that the runner's perception of comfort is heavily weighted by the properties of midsole thickness, cushioning, and resiliency as well as shoe fit.

#### Runner Assessment Approach and Recommendation

Methods to accurately assess shoe comfort are available (Mundermann et al., 2002; Lindorfer et al., 2019). Practically, there is no standardized method to assess this subjective measure other than asking the runner to rank and/or select based on individual preference.

#### Current Evidence

Two studies have explored the connection between comfort and biomechanical variability. The possible association between footwear comfort and biomechanical variability was postulated after findings in ergonomics literature. Sondergaard et al. (2010) found a positive correlation between measure of self-reported discomfort and degree of variability (measured by standard deviation) in center of pressure (COP) displacement during sitting tasks. The degree of biomechanical variability is also thought to contribute to running injury risk (Hamill et al., 2012) as some studies have found a connection between differences in coordination variability and injury populations (Heiderscheit et al., 2002; Miller et al., 2008; Seay et al., 2011). Meyer et al. (2017) calculated kinematic relative variability of foot motion (root mean square of the standard deviation/root mean square of the mean) from inertial sensor data. Thirty-six recreational runners performed trials in five different running shoes and ranked subjective comfort. The most and least comfortable shoes were compared. Only transverse plane angular velocity and frontal plane acceleration of the foot during the end of swing were significantly different between most and least comfortable shoe. Since these measurements represent transverse and frontal plane motions that are influenced by more proximal segments, the relationship to footwear is inconclusive. Lindorfer et al. (2020) examined coordination variability of lower extremity joint pairings in relation to most and least comfortable running shoes and did not find evidence to support this connection. Other than the seminal study by Mundermann et al. (2001) linking insole usage to injury reduction, no other studies have linked comfort and injury conclusively.

## Discussion

**Figure 4:**
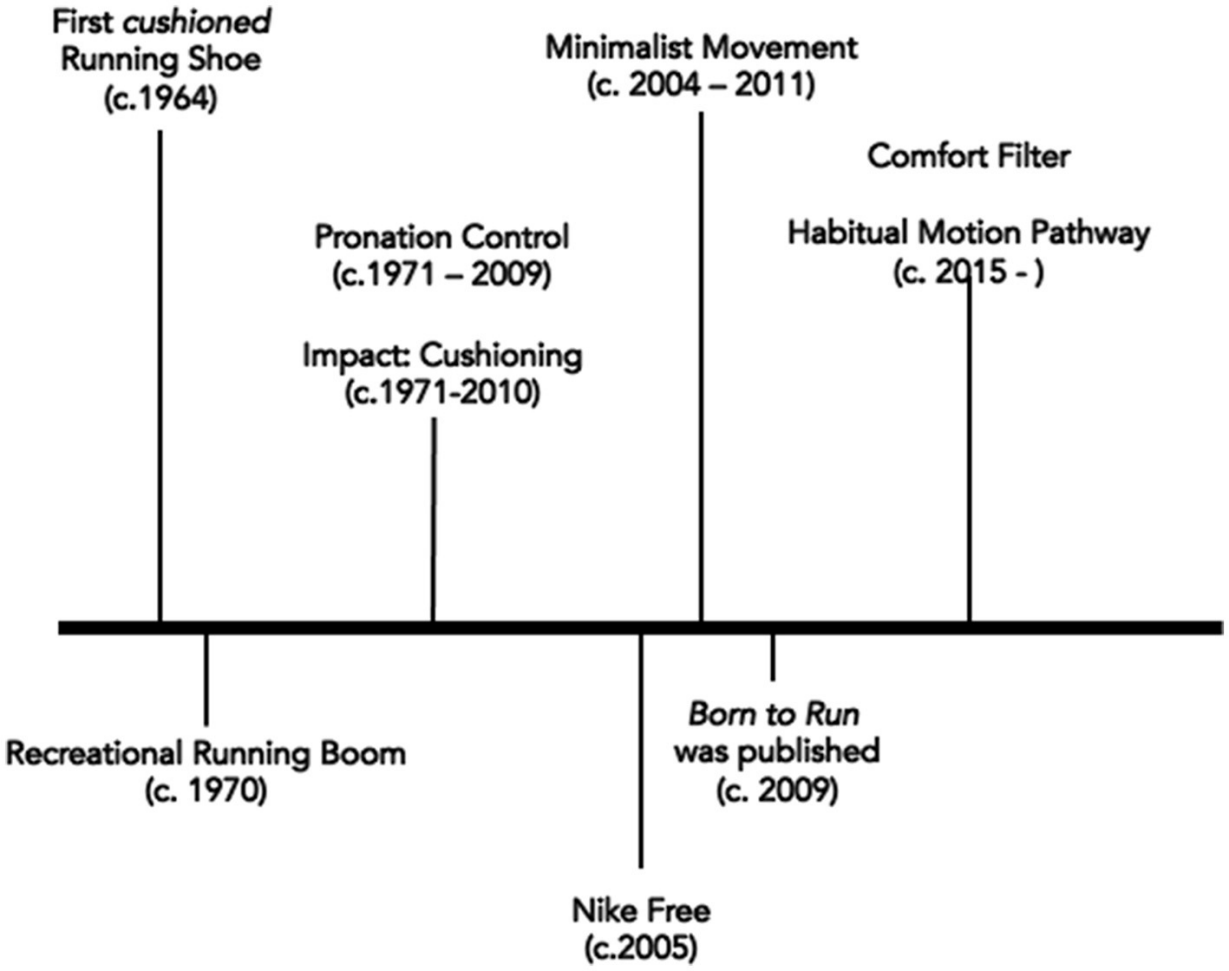
The four major injury paradigms in chronological order are described. Pronation control and Impact Force Modification are the older paradigms. Impact Force Modification was initially focused on cushioning and then later there was a minimalist movement influenced by the Nike Free running shoe as well as the book Born to Run. The two newer injury paradigms are Comfort Filter and Habitual Motion Pathway, popularized since the year 2015.

This review presents the four dominant running-related injury paradigms that have driven running footwear design and recommendation over the last half century. Importantly, our intention was to comprehensively describe the paradigms and not to advocate for one paradigm as the dominant model. We aimed to illustrate the common thread across the paradigms connecting the (1) identification of a biomechanical contribution to injury risk (rationale) which leads to (2) the design feature(s) to counteract the biomechanical risk factor(s) which, in turn, drives (3) the clinical assessment of the runner for the biomechanical factors to provide a recommendation for (4) footwear with the feature(s) to correct the biomechanical factor believed to be “at fault.” The chronology of injury-related paradigms illustrates the evolution of scientific thought on the biomechanical causes of running-related injury and the contributions shoes make to risk reduction (Figure 4). Pronation Control and Impact Force Modification are paradigms based on single and specific impairments or inputs to the human system, respectively. However, reducing running-related injuries by controlling pronation is not strongly supported by current evidence. Likewise, heavily cushioned running shoes do not consistently reduce the magnitude of vertical ground reaction forces experienced, nor do they seem to reduce injuries. Of note, shoes with very thick midsoles do seem to be more protective against running-related pain or injury than minimal-thickness shoes, especially for runners predisposed to foot and ankle issues.

From these paradigms, or perhaps due to their equivocal results on injury, Habitual Joint (Motion) Path and Comfort Filter emerged with a more complex, holistic, and individual-specific approach to matching footwear to runner. However, they remain largely untested to date. The Habitual Joint (Motion) Path is still in the early phases of research and findings from studies are not in definitive support. Similarly, while the Comfort Filter has a reasonable theoretical and research basis, very little has been done to directly test this paradigm for footwear recommendation. Moreover, both paradigms investigate biomechanical variability to assess a runner's response to footwear, but the type of biomechanical variability and its expected response should be clarified. Additionally, prospective studies are needed to understand the extent to which movement variability influences injury risk or performance outcomes.

A variety of measures of “variability” have been used to assess the latter two paradigms and each measure represents a different aspect of motor proficiency. For example, coordination variability and long-term correlations are measures of biological variability that describe how the system adapts and employs different motor control solutions to achieve the running task. Furthermore, coordination variability examines this flexibility at the local joint level, while long-term correlations examine it from a whole-body perspective. On the other hand, measures such as the standard deviation or coefficient of variation typically reflect endpoint variability, which is the consistency of a specific outcome (i.e., rearfoot angle at initial contact). As motor proficiency improves or optimizes, endpoint variability should reduce while biological variability should increase. Future research should take care to be deliberate in selecting assessment method, variability measure, hypothesis, and interpretation (Kimura et al., 2021) and implications for injury risk or performance outcomes.

Finally, we remind readers that research has yet to find consistent biomechanical risk factors for running-related injury (van der Worp et al., 2016; Ceyssens et al., 2019; Vannatta et al., 2020). Until those factors are found, it seems unrealistic to expect footwear to meaningfully reduce running-related injury risk. This is evidenced in our illustration of the paradigms. In many cases, the footwear feature was effective at its purpose (motion control shoes do reduce the amount of pronation and/or rearfoot eversion, cushioning does decrease in-shoe impact pressures, and one could argue recreational runners already self-select comfortable shoes). However, the rationale behind the paradigm seems misguided. Recent innovative research has called into question existing assumptions of the relation between commonly cited risky biomechanical factors, like high ground reaction forces and tissue strain (Matijevich et al., 2019). Moreover, the concept that there are global risk factors for injury (Davis and Futrell, 2016) or that one type of shoe could help all recreational runners may be inhibiting our ability to see the influence and role footwear may serve for each runner. Future research should be directed at examining the effect of specific footwear features on musculoskeletal tissue and place findings in context. That is, a description of the baseline tissue characteristics and specific running task demands are needed when explaining the influence of select footwear design. Additionally, we agree with other authors (Paquette and Miller, 2018) who have encouraged the need to monitor internal load and adaptation, particularly in response to novel footwear, to understand the relation between footwear and injury development.

### Performance Considerations

Up to this point, this review has focused on footwear paradigms relating to running injury. However, there is good evidence that specific features of running footwear can influence aspects of running performance. Thus, performance factors also should be considered when recommending running footwear, especially to elite or competitive runners who have specific performance goals. The footwear feature with the clearest link to performance is mass. Increasing shoe mass has been found to increase running time per distance (Hoogkamer et al., 2016) and energy consumption (Franz et al., 2012). For every 100 grams (3.5-oz) of added weight there is an ~1% increase in oxygen consumption (Frederick et al., 1984). It is important to note that the components of midsole construction—thickness, material, and additives—influence the mass of the shoe and thus, indirectly influence performance. The direct effects of these features and of midsole additives, such as carbon plates, on performance are less clear (Flores et al., 2021).

Data is conflicting about whether a carbon fiber plate improves running performance and if so, by what means (Roy and Stefanyshyn, 2006; Willwacher et al., 2014; Madden et al., 2016; Flores et al., 2019; Beck et al., 2020; Healey and Hoogkamer, 2020; Cigoja et al., 2021). Carbon fiber plates increase the longitudinal bending stiffness of the shoe, which has been found to reduce negative work at the metatarsophalangeal joint and alter the ground reaction force moment arm in a way that reduces oxygen consumption (Roy and Stefanyshyn, 2006; Willwacher et al., 2013, 2014; Stefanyshyn and Wannop, 2016). Indeed, the carbon fiber plate of the Nike Vaporfly shoe has been suggested as a key factor in the shoe's impressive 4% reduction in oxygen consumption. However, when Healey and Hoogkamer (2020) removed the effect of the carbon plate from the Vaporfly, they found no change in the oxygen consumption rate. Thus, the extent to which increased longitudinal bending stiffness via a carbon fiber plate can improve running performance needs further exploration (Ortega et al., 2021).

Other researchers have suggested that the shape (curved vs. flat) rather than the increased bending stiffness is what drives performance gains from a carbon fiber plate (Nigg et al., 2020). Specifically, some authors have argued for a “teeter-totter effect” in which a curved plate favorably shifts the ground reaction force vector anteriorly at push-off and creates a larger upward force on the heel (Nigg et al., 2021), which helps propulsion. This upward force helps to propel the foot off the ground, reducing the energetic cost of running. While early studies seem promising, these innovations need more testing. The optimal amount of longitudinal bending stiffness seems to be runner- and/or task-specific (Day and Hahn, 2020) as does the curvature of the plate (Nigg et al., 2020). However, a systematic method for assessing runners to customize these features does not currently exist.

Midsole thickness appears to affect running performance through several mechanisms. For example, too little thickness increases metabolic cost by requiring higher muscle activity to absorb impact forces (Tung et al., 2014). Indeed, there is a metabolic cost savings for adding underfoot cushioning. For example, shod running resulted in 3–4% lower oxygen consumption and metabolic power than barefoot running, even though shod running increased mass at the foot (Franz et al., 2012). Increased midsole thickness increases effective leg length, which could positively influence running performance (Burns and Tam, 2020). Increasing thickness also could improve comfort by increasing the amount of cushioning under the heel and allowing for greater midsole compliance (i.e., more deformation available before “bottoming out”) and resiliency (i.e., more material available to return energy). Conversely, too much thickness would not only unnecessarily increase mass but negatively influence running economy by increasing muscle activity to control movement in accessory planes (frontal, transverse) if the additional stack height creates instability (Hoogkamer, 2020).

It is important to note that the relation between the amount of midsole thickness and the material properties that lead to midsole resiliency are not well-established. Some studies have found improvements in running economy when running in footwear equipped with resilient midsole material compared with minimalist/maximalist footwear (Sinclair et al., 2016c), while others have not seen differences in energetic costs using these materials compared with conventional EVA foam (Flores et al., 2019). Studies of the Nike VaporFly and its successor models point to the high resiliency of the thick midsole, whose innovative materials keep the added mass low, as a main source of the significant performance gains associated with it (Hoogkamer et al., 2019). There is likely a “sweet spot” for midsole thickness for each runner, given the counteracting effects noted above (Hoogkamer, 2020). However, a method to determine that sweet spot remains elusive.

Finally, while comfort has been suggested to be relevant to injury risk reduction, it also may indicate the potential for improved performance, but results are inconclusive. While one study found a small (0.7%) improvement in running economy in the most (vs. least) comfortable shoe (Luo et al., 2009), a more recent study (Lindorfer et al., 2020) found no significant difference in oxygen consumption between the most comfortable shoe and the least comfortable shoe after running two 6-min running trials for each condition.

### Practical Implications

Extensive research has been conducted on the effects of running shoes on biomechanics. However, the fundamental practical question remains largely unanswered: how do we appropriately pair shoes to runners to optimize their experience and maximize their performance potential without increasing injury risk? Based on current evidence and in alignment with other investigators (Richards et al., 2009; Napier and Willy, 2018), the scientifically supported *general* recommendation to runners selecting footwear should be to pick the lightest and most comfortable shoe with the least amount of pronation control technology. Reducing shoe mass appears to be the only shoe feature that yields consistent results, that is, reduced energetic cost. While findings do not support the premise that biomechanics or running economy are significantly associated with comfort, it stands to reason that wearing a comfortable shoe likely does not have deleterious effects. Additionally, the current evidence suggests that medial arch technology or controlling pronation does not reduce injury as once believed.

Importantly, this recommendation for footwear may be scientifically correct but is likely ineffectual at optimizing shoe to runner because it relies on a small body of published research and on the *absence* of benefit from other recommendation strategies rather than benefits of a proposed strategy. Moreover, shoes are typically recommended to the runner without considering characteristics of the runner or running purpose (i.e., task demands and goals). We posit that context should be considered when proposing runner-specific footwear. Diversification of footwear based on type of runs (long run, speed work, race) and runner skill level, or functional groupings as Nigg suggests (Nigg et al., 2017), may increase the efficacy of certain shoe technologies. However, this idea needs to be rigorously tested as there is some contrasting evidence regarding the benefit (or harm) from parallel use of multiple running shoes (Malisoux et al., 2015; van der Worp et al., 2015).

In the absence of clear and global injury risk factors, running medicine clinicians should understand how footwear features could influence running biomechanics and/or musculoskeletal tissue and decide whether these design features would benefit their patient/athlete. We provide a few examples below:

1. Shoes with greater midsole thickness may provide more cushioning, which could be beneficial for a runner as it's perceived as more comfortable. However, the increase in thickness also increases the distance between foot and the ground (stack height) and could pose a risk of ankle sprain or peroneal tendonitis to runners who typically rearfoot strike but may not have the muscular strength or ankle stability to accommodate the increased challenge stack height brings. Additionally, increased stack height may lead to longer stride lengths and lower step rates, which in turn increases the loads experienced through the hip and hip musculature (Lenhart et al., 2014a) and may increase issues for runners who already have poor hip strength and/or neuromuscular control or hip pain.
2. Likewise, minimalist shoes may increase strain on posterior musculature either directly by a lower difference in forefoot-to-rearfoot midsole stack height (reduced heel-to-toe drop) or indirectly by increasing step rate. Minimalist shoes may be inappropriate for runners with acute posterior muscle strains and tendinitis but could be used, if dosed properly, to improve tendon stiffness and strength in chronic conditions.
3. New midsole foam material, like that found in the Nike Vaporfly, influences mechanical work and power at the ankle (Hoogkamer et al., 2019). Runners with acute Achilles tendonitis may benefit in the short-term from a reduction in ankle mechanical work or power. However, consistent use of this shoe long-term may degrade Achilles tendon stiffness and ability to effectively withstand strain, potentially setting multi-sport or multi-event runners up for injury in the future. Again, consideration of the purpose of training/racing and the tissue-level response to footwear feature becomes important for the recommendation. Runners susceptible to or with existing Achilles tendinopathy may benefit from reserving running in shoes with this type of midsole foam to racing only for the metabolic cost gains but train in shoes with different foam to build or maintain tendon stiffness.

## Limitations

We have limited this review to focus only on paradigms for shoe design and recommendation methods. As such, we did not report on the epidemiology of running-related injuries and the literature around reducing risk through gait retraining—either proactively or following injury—or strength training, namely foot strengthening. While these are important and inter-related topics, they call for their own reviews as they have a slightly different assumption (i.e., fix the runner not the shoe) than our review. We also limited our discussion of footwear features to those that directly related to each paradigm. For a comprehensive review of the influence of specific footwear features on biomechanics, please see (Hoitz et al., 2020; Sun et al., 2020).

## Conclusion

Practicing evidence-based running medicine relies on sound knowledge of basic shoe construction and the evolution of shoe design based on injury paradigms. Running medicine clinicians are encouraged to seek out new information regarding footwear construction and the possible implications for their patients. Likewise, we encourage footwear manufacturers to specify the intended purpose of footwear features and researchers to provide a more detailed and comprehensive description of sample groups so that clinicians can more appropriately recommend footwear to runners and interpret findings, respectively. To date, the available paradigms have very limited research evidence to support them. The best *general* recommendation based on the evidence available and considering the least likelihood of harm is to recommend a shoe that is lightweight, comfortable, and has as minimal pronation control technology as possible.

## Author Contributions

This study was conceptualized by CA. CG contributed to literature searches and compilation of evidence. CA, CG, and JZ contributed to manuscript writing. CA, JZ, CG, and MH contributed to revising and approving the final version of the manuscript. All authors contributed to the article and approved the submitted version.

## Conflict of Interest

CA and JZ both have received consulting fees from Diadora S.P.A. and have received research funding from adidas AG. The remaining authors declare that the research was conducted in the absence of any commercial or financial relationships that could be construed as a potential conflict of interest.

## Publisher's Note

All claims expressed in this article are solely those of the authors and do not necessarily represent those of their affiliated organizations, or those of the publisher, the editors and the reviewers. Any product that may be evaluated in this article, or claim that may be made by its manufacturer, is not guaranteed or endorsed by the publisher.
